# Characteristics of imported and domestic malaria cases in Gyeonggi Province, Korea

**DOI:** 10.4178/epih.e2024087

**Published:** 2024-11-08

**Authors:** Sunghee Hong, Jihye Kim, Soo-Nam Jo, Jong-Hun Kim, Boyoung Park, Bo Youl Choi

**Affiliations:** 1 Institute for Health and Society, Hanyang University, Seoul, Korea; 2 Department of Statistics and Data Science, Graduate School, Dongguk University, Seoul, Korea; 3 National Health Insurance Service, Wonju, Korea; 4 Gyeonggi Infectious Disease Control Center, Suwon, Korea; 5 Department of Social and Preventive Medicine, Sungkyunkwan University School of Medicine, Suwon, Korea; 6 Department of Preventive Medicine, Hanyang University College of Medicine, Seoul, Korea

**Keywords:** Malaria, Plasmodium vivax, Plasmodium malariae

## Abstract

**OBJECTIVES:**

This study explored 11 years of malaria data from mandatory reporting in Gyeonggi Province, Korea, to provide information for prevention strategies by linkage to nationwide health claims data.

**METHODS:**

Reported malaria cases in Gyeonggi Province from 2011 to 2021 were linked to medical usage data from the National Health Insurance Database. Data about hospitalization, antibiotic prescription and duration, malarial species, and sociodemographic information of the cases were included.

**RESULTS:**

Between 2011 and 2021, a total of 3,011 malaria cases were reported, consisting of 2,828 domestic (93.9%) and 183 imported (6.1%) cases. Over 80% of the cases involved males, with the majority of patients being in their 20s. Both domestic and imported cases peaked between June and August over the years. Imported cases had a higher hospitalization rate (66.9%) compared to domestically-acquired cases (54.9%). There was a significant variation in treatment rates, with 80.7% of imported cases and 74.6% of domestic cases receiving treatment. For domestic cases, chloroquine combined with primaquine was the most commonly prescribed treatment (77.0%), while atovaquone-proguanil was frequently used for imported cases (25.9%). *Plasmodium vivax* was the predominant species in domestic cases (94.9%), whereas *P. malariae* was more common in imported cases (62.3%). The overall number of reported malaria cases declined following a sharp decrease in imported cases in 2020 and 2021.

**CONCLUSIONS:**

Despite a decreasing trend in malaria cases reported in Gyeonggi Province, imported cases exhibited higher hospitalization rates and different antibiotic prescription and treatment patterns, reflecting the presence of a different malarial species.

## GRAPHICAL ABSTRACT

[Fig f4-epih-46-e2024087]

## Key Message

• Between 2011 and 2021, 94% of malaria cases reported in Gyeonggi Province were domestically acquired, with over 80% of cases involving male patients.

• Domestic cases were most common among individuals in their 20s, with *P. vivax* being the dominant species.

• Imported cases primarily involved *P. malariae*, which showed higher hospitalization and treatment rates.

## INTRODUCTION

Malaria is a severe, life-threatening disease transmitted to humans by mosquitoes. It manifests with symptoms such as fever, chills, rigors, and headaches. Respiratory symptoms include cough and runny nose, while digestive symptoms feature abdominal pain, indigestion, nausea, and jaundice [1]. The World Malaria Report indicates that there were 247 million cases of malaria globally in 2021, marking an increase from previous years, despite the ongoing coronavirus disease 2019 (COVID-19) pandemic [2].

In Korea, malaria was eradicated for a decade before cases resurged in 1994. Following a peak of 4,141 new cases in 2000, the incidence of malaria subsequently declined [1], and approximately 500 new cases have been reported annually since 2012 [3]. During the COVID-19 pandemic, the number of new malaria cases in Korea decreased, but there was a resurgence in 2022 and 2023 [3]. Malaria cases in Korea exhibit significant seasonal fluctuations, with increases from April to October. This period aligns with a rise in the mosquito population, which in turn leads to a higher prevalence of mosquito-borne diseases.

Malaria cases in Korea are broadly divided into imported and domestic cases. Although imported cases constitute a small proportion of the total, their numbers have been gradually increasing [1,3]. During the COVID-19 pandemic, there was a sharp decline in the number of imported cases; however, these numbers rebounded to pre-pandemic levels afterward. Imported cases exhibit distinct epidemiological and clinical characteristics based on the malaria species involved.

Previous studies on malaria infection have primarily relied on data reported from the national surveillance system or from individual hospitals and military services [4–6]. However, to fully understand the epidemiological and clinical characteristics of malaria, it is essential to link the national surveillance system, which encompasses 100% of diagnosed cases, with clinical data. Therefore, we investigated the epidemiological and clinical characteristics of malaria using linked data from region-wide reported malaria cases and nationwide health insurance data in Korea. We analyzed all cases collectively and then examined domestic and imported cases separately.

## MATERIALS AND METHODS

### Study settings and data sources

In Korea, infectious diseases that are highly contagious, have a poor prognosis, or lack preventive measures are classified as national notifiable infectious diseases. Medical providers are required to report cases of these diseases to regional health centers. The data are then reviewed by local health authorities and forwarded to the Korea Disease Control and Prevention Agency (KDCA) in accordance with the Infectious Disease Control and Prevention Act [7]. Malaria is included in the Mandatory Surveillance System, which mandates that cases be reported to a local public health center within 24 hours of diagnosis [7]. Thus, all diagnosed cases of malaria are captured by the Mandatory Surveillance System. To capture all reported malaria cases from the Mandatory Surveillance System in a specific region, we analyzed data from the Gyeonggi Infectious Diseases Control Center (GIDCC) spanning from 2011 to 2021. Gyeonggi Province is situated in the central-western part of the Korean Peninsula and is home to 13.93 million people, representing 27.0% of the total population of Korea. This region covers an area of 10,195 km^2^, which constitutes 10.1% of Korea’s national territory, as of 2021 [8].

For the analysis of the clinical characteristics of malaria cases, the National Health Insurance Service (NHIS)–National Health Information Database (NHID) was linked to reported malaria cases using resident registration numbers from January 1, 2011 to December 31, 2021. The NHIS operates a universal and mandatory health insurance system that encompasses all residents of Korea. It offers a range of benefits including diagnosis, treatment, rehabilitation, and support for birth and death, as well as health promotion activities such as general health and cancer screenings. Consequently, the NHID holds extensive nationwide data on healthcare utilization and prescriptions [9], which are pertinent to the clinical characteristics under study.

### Socio-demographic and clinical factors

In 2000, 17 districts, including Incheon, Gyeonggi, and the northern part of Gangwon, were designated as high-risk regions for malaria [10]. By 2015, Paju-si, Yeoncheon-gun, Gimpo-si, and Goyang-si in Gyeonggi Province, primarily located near the border with North Korea, were identified as areas with high malaria incidence [11,12].

In the GIDCC dataset, the variable indicating the region of infection was categorized as either “domestic” or “imported.” Socio-demographic variables, including sex and age, were also considered, with age being divided into 10-year intervals. The variable for army service was classified as either “completed army service” or “currently in army service.” The malarial species variable was identified as one of the following: *Plasmodium falciparum*, *P. vivax*, *P. malariae*, or *P. ovale*. Additionally, in the GIDCC data, hospitalization status was specified as either “hospitalization” or “outpatient.” In the NHIS-NHID dataset, detailed information on malaria treatments, including the history of hospital visits and prescriptions, is available. We analyzed the prescription records and the number of hospital visits associated with the malaria disease code. Following clinical guidelines, we categorized the specified list of drugs into the following groups: chloroquine and primaquine; atovaquone-proguanil/pyronaridine-artesunate/mefloquine and primaquine; mefloquine and primaquine; atovaquone-proguanil; pyronaridine-artesunate; mefloquine; atovaquone-proguanil/mefloquine and pyronaridine-artesunate; as well as other commonly used medications [10].

### Study participants

The GIDCC data included 3,062 cases of malaria in Gyeonggi Province from 2011 to 2021. Of these, 3,011 patients had linked NHIS records and medical documentation related to malaria. The cases were categorized as either domestic or imported, depending on the source of infection. All reported malaria cases in this study occurred in Gyeonggi Province. Cases with a suspected infection location within Korea were classified as domestic, while those suspected to have been contracted outside of Korea (overseas) were classified as imported. In terms of hospitalization, the analysis did not consider the duration of the hospital stay; it only noted whether the patients were hospitalized or treated on an outpatient basis.

For clinical and treatment characteristics, the prescribed medications for malaria and the number of hospital visits coded for malaria were determined by linking the NHIS-NHID data with the GIDCC data. Patients who were in military service were excluded if the treatments administered in military hospitals could not be identified in the NHIS-NHID data.

### Statistical analysis

The baseline characteristics of patients with malaria included in the study were presented as numbers and percentages, divided into domestic and imported cases. We then analyzed the prescribed medications for malaria and the number of hospital visits coded for malaria, excluding patients in military service, and categorized these into domestic and imported cases. Differences between the 2 groups were assessed using chi-square analysis or t-tests. Additionally, the prescribed medications were detailed according to the species of malaria. Statistical analyses were conducted using R version 4.2.2 (R Foundation for Statistical Computing, Vienna, Austria).

### Ethics statement

This research received approval from the Institutional Review Board of Hanyang University (approval No. HYUIRB-202206-003-2). The requirement for informed consent was waived due to the anonymization of individual identities prior to database construction.

## RESULTS

**Figure 1:**
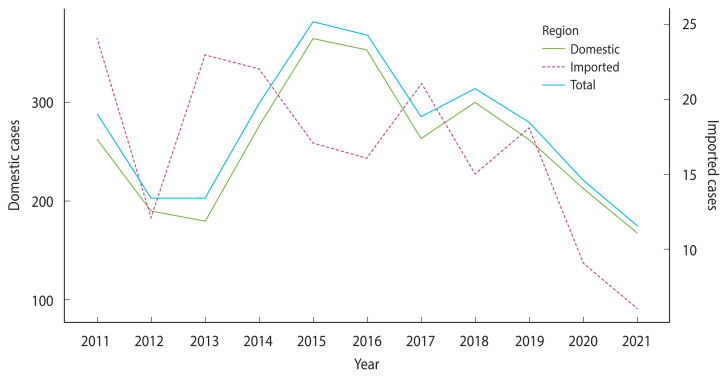
Count of malaria cases by year.

The total number of patients with malaria is shown in Figure 1, stratified by infection source per year. The incidence of domestic cases reached its peak in 2015, followed by a decline, while the number of imported cases has been decreasing since 2013, albeit with significant fluctuations. Notably, during the COVID-19 pandemic in 2020 and 2021, the incidence of imported cases significantly decreased.

**Figure 2:**
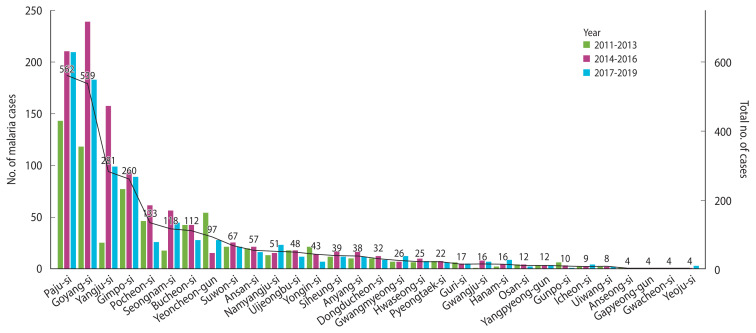
Number of malaria cases in Gyeonggi Province by year.

When considering the number of patients by region over 3-year intervals, Paju-si recorded the highest number of cases, followed by Goyang-si, Yangju-si, and Gimpo-si. Regions with fewer than 100 cases included Yangpyeong, Gapyeong, and Yeoju. Notably, there was a significant increase in the overall number of patients across the entire region during 2015–2016, with a particularly sharp rise observed in Yangju (Figure 2).

**Figure 3:**
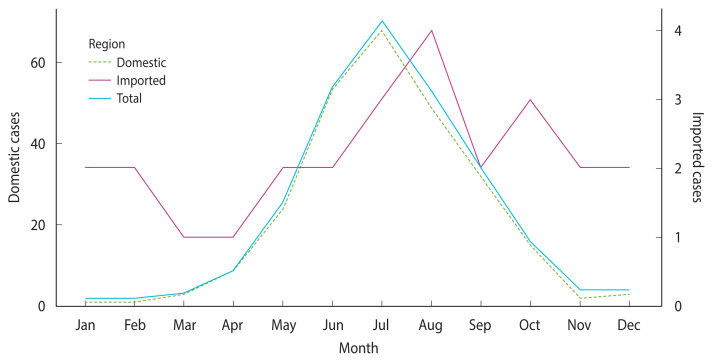
Number of malaria cases by month (2011–2021).

The average monthly case data (Figure 3) indicate that domestic cases began to rise in April, peaked in July, and subsequently showed a declining trend. In contrast, imported cases fluctuated with alternating increases and decreases until they began to rise in June, reached a peak in August, and then continued to increase, stabilizing between September and November.

The baseline characteristics of patients with malaria in Gyeonggi Province from 2011 to 2021 are detailed in Table 1, categorized by origin as either domestic or imported. Over 80% of cases in both categories were male, with 82.6% in domestic cases and 83.1% in imported cases. The majority of domestic patients were in their 20s (42.1%), whereas the ages of imported patients were more evenly spread from their 20s to 50s (p<0.001). Regarding army service status, 26.1% of domestic patients were serving in the army at the time of diagnosis, in contrast to the imported group, which primarily consisted of civilians. The predominant malaria species among domestic patients was *P. vivax* (94.9%). In contrast, imported cases showed a higher prevalence of *P. falciparum* (62.3%), with *P. vivax* making up 27.9%. The species distribution of malaria significantly differed between domestic and imported patients (p<0.001). Considering the origin of the malaria species in imported cases, most cases suspected to have been contracted in Asia were due to *P. vivax*, while *P. falciparum* was the predominant species in other continents. Notably, West Africa was responsible for over half of the *P. malariae* infections.

When data from the GIDCC concerning malaria patients was linked to their treatment histories, it was found that over 70% of patients who had served in the army could not be matched with NHIS-NHID data. This discrepancy arose because treatment histories from military hospitals were not recorded in the NHIS-NHID. Consequently, the detailed treatment histories analyzed from the NHIS-NHID excluded military personnel. Table 2 displays the clinical and treatment characteristics of malaria cases, categorized by the source of infection. The hospitalization rates for domestic and imported malaria patients were 54.9% and 66.9%, respectively (p=0.001). Outpatient treatment was more common among domestic cases than imported ones, with rates of 39.9% versus 25.4%. Additionally, a higher percentage of imported patients received treatment compared to domestic patients (80.7 vs. 74.6%, p=0.07). Treatment regimens also differed; chloroquine and primaquine were more frequently used in domestic patients (77.0%), while atovaquone-proguanil was more common among imported patients (25.9%, p<0.001). Regarding hospital visits, 62.9% of domestic patients and 65.8% of imported cases had 1 recorded visit for malaria treatment (p=0.636).

Table 3 presents the treatment regimens based on the species of malaria. Among the 1,563 patients diagnosed with *P. vivax*, 76.3% were treated with both chloroquine and primaquine, following the malaria treatment guidelines. Approximately 20% were treated with either chloroquine or primaquine alone. For those infected with *P. falciparum*, all of whom had imported cases, the 3 most commonly prescribed treatments were atovaquone-proguanil, mefloquine, and a combination of pyronaridine-artesunate and mefloquine, which together accounted for 67.4% of the prescriptions. Around 80% of the patients with *P. falciparum* received treatment in accordance with the recommended guidelines.

## DISCUSSION

Over the past 11 years, there has been a decline in the number of malaria cases in Gyeonggi Province in Korea. Both domestic and imported cases have decreased; however, the reduction in imported cases was particularly notable during the COVID-19 pandemic due to the containment policies in place [13]. Among the malaria cases, more than 80% were male, with domestic cases comprising approximately 94%. The domestic cases predominantly involved younger individuals, with the highest proportion of patients aged between 20 years and 29 years. *P. vivax* was the most common malarial species in domestic cases, accounting for 94.9%, while *P. malariae* was more prevalent among imported cases, at 62.3%. Excluding military personnel, imported patients had higher rates of hospitalization (66.9%) and treatment (80.7%) compared to domestic patients, who had rates of 54.9% and 74.6%, respectively. Furthermore, there were significant differences in antibiotic prescription patterns between the 2 groups, with 80% of patients receiving prescriptions for medications appropriate for the specific type of malaria species. This reflects adherence to clinical guidelines for treatment regimens in both domestic and imported cases [14].

In addition to Gyeonggi Province, most other regions in Korea, with the exception of Gwangju, have experienced a decline in malaria cases [1]. Malaria is transmitted by mosquitoes carrying the parasite, and its incidence is influenced by several factors, including climate change, the habitat preferences of vector mosquitoes, and the behavioral characteristics of the population [15]. With the advent of global warming and an increase in outdoor activities, a rise in the incidence of various infectious diseases is anticipated. In response, Korea has implemented comprehensive strategies to control malaria. These include mass chemoprophylaxis among military personnel to prevent the spread of malaria to civilians. As malaria control programs have evolved, the focus of interventions has shifted to treating individual patients. Recently, the availability of rapid diagnostic test kits has made it possible to diagnose malaria within 5 days of the onset of fever [16]. This method quickly confirms infections, allowing for prompt treatment and effective management of patients. Early diagnosis not only helps prevent the further spread of malaria but also optimizes the use of healthcare resources, playing a crucial role in the success of Korea’s malaria control efforts.

*P. vivax* has long been an endemic malaria species in Korea [17]. It accounts for 94.9% of domestically infected malaria cases, particularly in Gyeonggi Province. Additionally, *P. vivax* is responsible for most malaria cases in Asia and the Americas, making it the second most prevalent species worldwide [18]. *P. vivax* was one of the major causes of casualties in the Korean army and United Nations forces during the Korean War. In 1993, *P. vivax* re-emerged, with the first case identified in a soldier in the demilitarized zone [19]. Subsequently, the occurrence of *P. vivax* malaria, especially in its initial phases, was predominantly documented among soldiers deployed near the demilitarized zone in Gyeonggi Province and northern Gangwon Province [20]. This pattern implies that North Korea could be a significant reservoir for *P. vivax* malaria, given its proximity to these regions. Herein, approximately one-third of domestic cases were associated with army service.

However, *P. malariae* accounted for 62.3% of imported malaria cases, followed by *P. vivax* at 29%, indicating a stark difference between domestic and imported infections. *P. malariae* is a less-studied species, and malaria caused by this species is referred to as “benign malaria” because of its low prevalence and mild clinical symptoms. *P. malariae* is prevalent in sub-Saharan Africa [21]. In this study, among 114 cases of *P. malariae*, 112 contracted the infection in Africa, reflecting its global distribution. *P. malariae* has become common in the areas where the incidence of *P. falciparum* has decreaed because of successful interventions [21]; therefore, successful interventions with other malarial species may increase the incidence of other rare malarial species. Despite the lower pathogenicity of *P. malariae*, which accounted for the majority of imported cases, 67.2% of individuals infected overseas were hospitalized, and 80% received treatment—rates significantly higher than those observed in domestic cases. This discrepancy may be attributed to the older age of patients with imported malaria compared to those with domestic malaria. Additionally, given that *P. malariae* is the most frequent coinfection in populations endemic for *P. falciparum* and *P. vivax*, the high proportion of *P. malariae* among imported cases, combined with the predominance of *P. vivax* in domestic cases, could pose a public health challenge in Korea. The variation in antibiotic regimens between domestic and imported cases may also be due to the different malaria species prevalent in these groups.

This study had several limitations. First, the treatment history of military personnel with malaria is reported weekly through notifications from the Korean Ministry of National Defense to the KDCA, which differs from the claims data in the NHID. Particularly among those infected domestically, the significant number of military personnel may introduce potential uncertainties and disparities in their information compared to the claims data in the NHID [22]. The treatment history relies on claims data from the NHID, suggesting the possibility that more patients may have received treatment than recorded. Therefore, it is necessary to verify and manage prescription records for these cases to ensure accuracy. Second, *P. vivax* infection can become severe or fatal and is commonly linked to a range of complications in non-immune adults [23]. Despite efforts in this study to connect the claims data in the NHID, establishing a clear association with malaria proved challenging. Additionally, most cases did not result in fatalities, and for those few cases where death occurred, with various time frames from malaria diagnosis to death, the mortality or fatality rate due to malaria could not be estimated. Third, in Korea, an estimated 50% of patients with *P. vivax* malaria have a prolonged incubation period [24]. Previous studies have identified factors influencing the length of the incubation period in *P. vivax* malaria, such as the location and timing of infection, as well as the onset of symptoms [24]. However, the unavailability of this information in our study precluded the assessment of such correlations. Fourth, our inability to confirm antimalarial drug resistance directly should be considered. Since the report of a low prevalence of chloroquine-resistant *P. vivax* strains (less than 0.5%; 2 out of 484) [25], there have been no further reports of malaria drug resistance in Korea. Additionally, in most foreign countries, there have been no drug resistance problems, except in Indonesia, Myanmar, Vietnam, Papua New Guinea, and India, which have reported some resistant cases [1]. Thus, we expected issues related to drug-resistant malaria with regard to imported cases would be minimal. Fifth, through this study, we identified that the treatment history of 62.2% of reported malaria cases (1,873 out of 3,011) was captured in the NHIS-NHID. Because the NHIS-NHID does not include claims from military hospitals, overseas treatments, treatments performed in public health centers, and treatments not covered by the NHIS in Korea, the NHIS-NHID alone has limitations in identifying all treatments. When we compared the presence of treatment history from the NHIS-NHID according to army service status, the treatment history for 74.1% of civilians, 84.0% of people who had finished army service, and 22.7% of people who were currently in army service could be identified. We can be certain that the treatment history of people who were in active army service was not captured well by the NHIS. Therefore, efforts to identify all treatment histories that could not be identified in the NHIS-NHID would be necessary to fully characterize malaria in Korea. Sixth, inappropriate doses of antimalarial drugs, considering patient weight and associated relapse, have been identified as an obstacle to malaria eradication [26]. Although anthropometric information is available in the Health Checkup database, information on patients who did not receive national health checkups was unavailable. In addition, owing to varying intervals between health checkups and malaria treatment, we were not able to determine whether the anthropometric information had a relation to the time of malaria infection or treatment. Thus, we could not consider weight in this study. Seventh, a domestic study revealed that among 1,546 malaria follow-up cases, 10 showed signs of recrudescence [27]. However, the NHIS-NHID lacked disease codes for recrudescence; therefore, this study did not take into account malaria recrudescence and reinfection. Further investigation into the characteristics and clinical details of reinfection cases is necessary.

Despite these limitations, our findings offer crucial insights into alternative strategies for preventing domestic malaria transmission and addressing cases introduced from abroad. This analysis leverages 11 years of linked data from the notification and health care insurance systems.

## Figures and Tables

**Figure f4-epih-46-e2024087:**
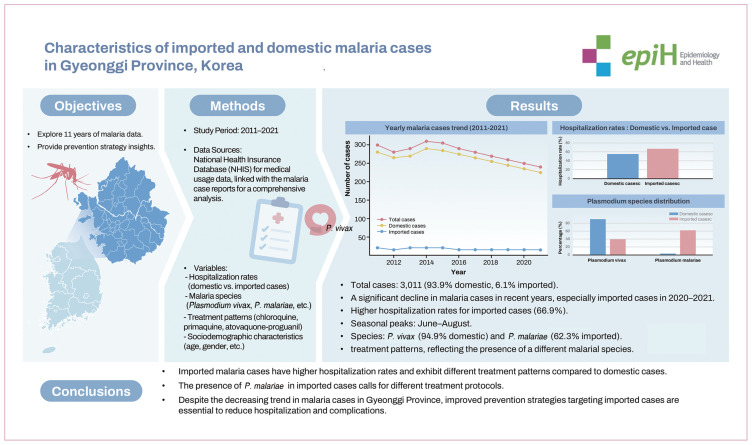


**Table 1:** Characteristics of domestic and imported malaria patients in Gyeonggi Province, 2011–2021 from the Gyeonggi Infectious Diseases Control Center data

Characteristics	Domestic	Imported	p-value
Total	2,828 (100)	183 (100)	
Sex			0.865
Male	2,335 (82.6)	52 (83.1)	
Female	493 (17.4)	31 (16.9)	
Age (yr)			<0.001
Mean±SD	36.1±16.7	41.9±14.4	
<10	23 (0.8)	0 (0.0)	
10–19	148 (5.2)	10 (5.5)	
20–29	1,190 (42.1)	36 (19.7)	
30–39	345 (12.2)	31 (16.9)	
40–49	435 (15.4)	51 (27.9)	
50–59	396 (14.0)	35 (19.1)	
60–69	189 (6.7)	15 (8.2)	
70–79	76 (2.7)	4 (2.2)	
80–89	23 (0.8)	1 (0.5)	
≥90	3 (0.1)	0 (0.0)	
Army service			<0.001
None	1,818 (64.3)	179 (97.8)	
Completed army service	250 (8.8)	0 (0.0)	
Currently in army service	738 (26.1)	2 (1.1)^1^	
Unknown	22 (0.8)	2 (1.1)	
Malaria species			<0.001
*P. falciparum*	0 (0.0)	114 (62.3)	
*P. vivax*	2,684 (94.9)	51 (27.9)	
*P. malariae*	0 (0.0)	1 (0.5)	
*P. ovale*	0 (0.0)	5 (2.7)	
Unknown	144 (5.1)	12 (6.5)	

Values are presented as number (%).

SD, standard deviation; *P*., *Plasmodium*.

1 Two patients who were in army service were infected from South Sudan.

**Table 2:** Clinical and treatment characteristics of domestic and imported malaria patients in Gyeonggi Province, 2011–2021, by linking data from the Gyeonggi Infectious Diseases Control Center with the NHIS-NHID^1^

Characteristics	Domestic	Imported	p-value
Total	2,090 (100)	181 (100)	
Hospitalization			0.001
Hospitalization	1,148 (54.9)	121 (66.9)	
Outpatient	834 (39.9)	46 (25.4)	
Others	108 (5.2)	14 (7.7)	
Treatment			0.070
Never	531 (25.4)	35 (19.3)	
Ever	1,559 (74.6)	146 (80.7)	
Treatment regimen			<0.001
Chloroquine and primaquine	1,200 (77.0)	26 (17.8)	
Primaquine	207 (13.3)	7 (4.8)	
Chloroquine	105 (6.7)	7 (4.8)	
Mefloquine and primaquine	11 (0.7)	8 (5.5)	
Mefloquine	5 (0.3)	23 (15.8)	
Atovaquone-proguanil	0 (0.0)	38 (25.9)	
Pyronaridine-artesunate	0 (0.0)	3 (2.0)	
Atovaquone-proguanil and primaquine	5 (0.3)	7 (5.4)	
Pyronaridine-artesunate and primaquine	4 (0.3)	1 (0.7)	
Pyronaridine-artesunate and mefloquine	1 (0.1)	0 (0.0)	
Pyronaridine-artesunate and atovaquone-proguanil	0 (0.0)	1 (0.7)	
Others	21 (1.3)	25 (17.1)	
No. of hospital visits for malaria treatment		0.767	
Mean±SD	1.20±1.18	1.23±1.03	
1	980 (62.9)	96 (65.8)	0.636
2	420 (26.9)	36 (24.7)	
3	93 (6.0)	6 (4.1)	
>3	66 (4.2)	8 (5.5)	

Values are presented as number (%).

NHIS-NHID, National Health Insurance Service–National Health Information Databas; SD, standard deviation.

1 Of the 2,828 domestic and 183 imported cases, 739 and 2 patients who were in army service were excluded because data from more than 75% of them could not linked from the NHIS-NHID, possibly owing to receiving treatment in military hospitals.

**Table 3:** Malaria species-specific treatment regimen in Gyeonggi Province, 2011–2021^1^

Treatment regimen	Malaria species		
	*P. falciparum*	*P. vivax*	Others
Total	86 (100)	1,563 (100)	56 (100)
Chloroquine and primaquine	0 (0.0)	1,193 (76.3)	33 (58.9)
Chloroquine	2 (2.3)	100 (6.4)	10 (17.9)
Primaquine	3 (3.5)	209 (13.4)	2 (3.6)
Atovaquone-proguanil and primaquine	2 (2.3)	9 (0.6)	1 (1.8)
Pyronaridine-artesunate and primaquine	0 (0.0)	4 (0.3)	1 (1.8)
Mefloquine and primaquine	2 (2.3)	15 (1.0)	2 (3.6)
Atovaquone-proguanil	31 (36.0)	4 (0.3)	3 (5.4)
Pyronaridine-artesunate	3 (3.5)	0 (0.0)	0 (0.0)
Mefloquine	20 (23.3)	6 (0.4)	2 (3.6)
Pyronaridine-artesunate and atovaquone-proguanil	2 (2.3)	0 (0.0)	0 (0.0)
Pyronaridine-artesunate and mefloquine	7 (8.1)	0 (0.0)	0 (0.0)
Others	14 (16.3)	23 (1.5)	2 (3.6)

Values are presented as number (%).

*P*., *Plasmodium*.

1 Of the 2,828 domestic and 183 imported cases, 739 and 2 patients who were in army service were excluded; In addition, those without treatment information in the National Health Insurance Service–National Health Information Database were excluded.
