# Characteristics of imported and domestic malaria cases in Gyeonggi Province, Korea

**DOI:** 10.4178/epih.e2024087.E

**Published:** 2025-03-17

**Authors:** Sunghee Hong, Jihye Kim, Soo-Nam Jo, Jong-Hun Kim, Boyoung Park, Bo Youl Choi

**Affiliations:** 1 Institute for Health and Society, Hanyang University, Seoul, Korea; 2 Department of Statistics and Data Science, Graduate School, Dongguk University, Seoul, Korea; 3 National Health Insurance Service, Wonju, Korea; 4 Gyeonggi Infectious Disease Control Center, Suwon, Korea; 5 Department of Social and Preventive Medicine, Sungkyunkwan University School of Medicine, Suwon, Korea; 6 Department of Preventive Medicine, Hanyang University College of Medicine, Seoul, Korea

To the Editor:

The authors regret that there were errors in the text.

Content of correction:

Imported cases’ major malaria species is *P. falciparum* (62.3%), not *P. malariae*.

So, *P. malariae* in the graphical abstract key message, keywords in the abstract, results, and discussion is changed to *P. falciparum*.

*Plasmodium vivax* was the predominant species in domestic cases (94.9%), whereas *P. malariae* was more common in imported cases (62.3%).

*Plasmodium vivax* was the predominant species in domestic cases (94.9%), whereas *P. falciparum* was more common in imported cases (62.3%). (Revised)

KEY WORDS: Malaria, *Plasmodium vivax*, *Plasmodium malariae*

KEY WORDS: Malaria, *Plasmodium vivax*, *Plasmodium falciparum* (Revised)

Notably, West Africa was responsible for over half of the *P. malariae* infections.

Notably, West Africa was responsible for over half of the *P. falciparum* infections. (Revised)

*P. vivax* was the most common malarial species in domestic cases, accounting for 94.9%, while *P. malariae* was more prevalent among imported cases, at 62.3%.

*P. vivax* was the most common malarial species in domestic cases, accounting for 94.9%, while *P. falciparum* was more prevalent among imported cases, at 62.3%. (Revised)

However, *P. malariae* accounted for 62.3% of imported malaria. cases, followed by *P. vivax* at 29%, indicating a stark However, *P. malariae* accounted for 62.3% of imported malaria cases, followed by *P. vivax* at 29%, indicating a stark difference between domestic and imported infections. *P. malariae* is a less-studied species, and malaria caused by this species is referred to as “benign malaria” because of its low prevalence and mild clinical symptoms. *P. malariae* is prevalent in sub-Saharan Africa [21].

In this study, among 114 cases of *P. malariae*, 112 contracted the infection in Africa, reflecting its global distribution. *P. malariae* has become common in the areas where the incidence of *P. falciparum* has decreased because of successful interventions [21]; therefore, successful interventions with other malarial species may increase the incidence of other rare malarial species. Despite the lower pathogenicity of *P. malariae*, which accounted for the majority of imported cases, 67.2% of individuals infected overseas were hospitalized, and 80% received treatment—rates significantly higher than those observed in domestic cases. This discrepancy may be attributed to the older age of patients with imported malaria compared to those with domestic malaria.

Additionally, given that *P. malariae* is the most frequent coinfection in populations endemic for *P. falciparum* and *P. vivax*, the high proportion of *P. malariae* among imported cases, combined with the predominance of *P. vivax* in domestic cases, could pose a public health challenge in Korea.

However, *P. falciparum* accounted for 62.3% of imported malaria cases, followed by *P. vivax* at 29%, indicating a stark difference between domestic and imported infections. Additionally, *P. malariae* is a less-studied species due to its low parasite density and frequent co-infections with *P. falciparum*, making its detection challenging with routine diagnostic methods. Although *P. malariae* is often considered to cause mild disease, it can persist as a chronic infection and has been associated with nephropathy. This species is prevalent in sub-Saharan Africa, with reported prevalence ranging from 0% to 32% depending on diagnostic methods, and seroprevalence reaching up to 56% in some countries [21].

In this study, among 114 cases of *P. falciparum*, 112 were acquired in Africa, reflecting its global distribution. Meanwhile, although *P. malariae* remains relatively rare, its prevalence may have increased in regions where *P. falciparum* has declined due to successful interventions [21]. Therefore, successful interventions with other malarial species may increase the incidence of other rare malarial species.

Given the high proportion of *P. falciparum* among imported cases, which accounted for 62.3% of infections, 67.2% of individuals infected overseas were hospitalized, and 80% received treatment—rates significantly higher than those observed in domestic cases. This discrepancy may be attributed to the severe nature of *P. falciparum* infections and the older age of patients with imported malaria compared to those with domestic malaria.

Additionally, *P. malariae* is commonly detected as a coinfection in populations endemic for *P. falciparum* and *P. vivax*. The high proportion of *P. falciparum* among imported cases, combined with the predominance of *P. vivax* in domestic cases, could pose a public health challenge in Korea. (Revised)

DOI of original article https://doi.org/10.4178/epih.e2024087

